# Wearable Near-Eye Tracking Technologies for Health: A Review

**DOI:** 10.3390/bioengineering11070738

**Published:** 2024-07-22

**Authors:** Lisen Zhu, Jianan Chen, Huixin Yang, Xinkai Zhou, Qihang Gao, Rui Loureiro, Shuo Gao, Hubin Zhao

**Affiliations:** 1 HUB of Intelligent Neuro-Engineering, Aspire CREATe, IOMS, Division of Surgery and Interventional Science, University College London, London HA7 4LP, UK; lisen.zhu@outlook.com (L.Z.); jianan.chen.22@ucl.ac.uk (J.C.); huixinyang@buaa.edu.cn (H.Y.); xinkai.zhou.21@uc.ac.uk (X.Z.); r.loureiro@ucl.ac.uk (R.L.); 2 School of Instrumentation and Optoelectronics Engineering, Beihang University, Beijing 100191, China; qihanggao@buaa.edu.cn

**Keywords:** near eye tracking, video oculography, infrared oculography, electrooculography

## Abstract

With the rapid advancement of computer vision, machine learning, and consumer electronics, eye tracking has emerged as a topic of increasing interest in recent years. It plays a key role across diverse domains including human–computer interaction, virtual reality, and clinical and healthcare applications. Near-eye tracking (NET) has recently been developed to possess encouraging features such as wearability, affordability, and interactivity. These features have drawn considerable attention in the health domain, as NET provides accessible solutions for long-term and continuous health monitoring and a comfortable and interactive user interface. Herein, this work offers an inaugural concise review of NET for health, encompassing approximately 70 related articles published over the past two decades and supplemented by an in-depth examination of 30 literatures from the preceding five years. This paper provides a concise analysis of health-related NET technologies from aspects of technical specifications, data processing workflows, and the practical advantages and limitations. In addition, the specific applications of NET are introduced and compared, revealing that NET is fairly influencing our lives and providing significant convenience in daily routines. Lastly, we summarize the current outcomes of NET and highlight the limitations.

## 1. Introduction

Tracking the eye gaze shows great significance in various fields, such as human–computer interaction (HCI) [1], virtual reality (VR) [2,3], driver monitoring systems [4,5], and clinical studies [6,7,8,9], and there have been decades of evolvement in the eye-gaze-tracking techniques. Based on the distance between the camera and the user, eye-tracking technologies can be categorized into remote (>10 cm) and near-eye tracking (NET) (<10 cm) scenarios. In remote settings, the images are generally captured by cameras or webcams, requiring analysis of the eye region [10] or whole face region [11]. Conversely, NET settings involve focusing solely on the eye region and capturing eye movement by glasses or head-mounted devices. NET devices are typically fixed to the eyes and capture movements at close range. This setup minimizes the impact of head movements and environmental changes. In contrast, remote eye tracking involves extracting the eye gaze from complex backgrounds and managing variations in head pose. Furthermore, NET devices generally allow free movement for users [12]. However, remote eye tracking often limits the activity of users, such as requiring users to sit in front of the camera [12]. Although head-mounted NET systems are more intrusive, they offer superior accuracy compared to remote video-based techniques [13]. Consequently, these features make NET potentially more feasible for translational applications such as stroke assessment [14] and surgical assistance [15], especially those integrated into VR or augmented reality (AR) systems [16].

The techniques for NET have evolved from early invasive stick pointers [17] and scleral search coil (SSC) [18] to non-invasive approaches such as electrooculography (EOG) [19], infrared oculography (IOG or IROG), and video oculography (VOG). Table 1 shows a comparison of these eye-tracking methods from the aspects of cost, wearability, invasiveness, and accuracy. Non-invasive methods greatly eliminate the requirement for specialized preparation and devices and reduce the related risks and discomfort for the users.

Among these non-invasive NET methods, EOG has high robustness and low power consumption, making it suitable for long-term health monitoring devices [20]. IOG provides high-precision and detailed eye movement data, enabling the possible capability for diagnosing and regular monitoring of neurological diseases [21]. However, high-quality IOG infrared cameras and relevant sensors are expensive. In contrast, VOG achieves a good balance between cost and performance, and it is suitable for evaluating eye movement and recording eye appearance [22].

**Table 1:** Comparison of typical eye-tracking methods [17,23].

	Cost	Portability/Wearability	Invasiveness	Accuracy
Stick pointer	Low-cost	Portable	Invasive	Lower
SSC	Expensive	Benchtop	Invasive	High
EOG	Moderate cost	Portable	Non-invasive	High
IOG	Moderate to high-cost	Portable	Non-invasive	High
VOG	Moderate cost	Wearable	Non-invasive	High

Several literature reviews on NET have been published in recent years [24,25,26,27,28,29,30,31,32]. They provided a comprehensive understanding of eye tracking (ET) technology in diverse aspects, such as attentional research [24], VR [25], information selection [26], emotion recognition [27,28], consumer platforms [29], etc. Particularly, several review papers provide detailed and thorough insight into health-related disciplines, covering endoscopy [30], surgical research [31], and radiological image interpretation [32]. Despite the continuous development and use of NET in health-related domains, there is a lack of a dedicated review that can cover the current progress and application of NET for health. 

**Figure 1:**
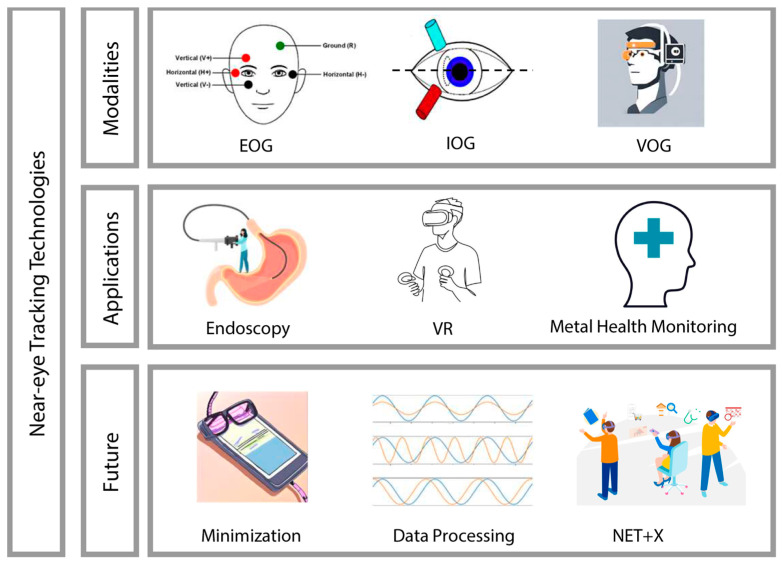
Overview of wearable near-eye tracking technologies for health.

Therefore, this review synthesizes articles on health-related NET and provides a detailed overview of its technology, applications, as well as future directions. To conduct this review, we meticulously searched major academic databases, including PubMed, IEEE Xplore, and Google Scholar. Our search strategy employed a combination of keywords including: (“near eye-tracking” OR “NET”) AND (“wearable technology” OR “wearable devices” OR “health monitoring”) AND (“video oculography” OR “VOG” OR “infrared oculography” OR “IOG” OR “electrooculography” OR “EOG” OR “eye movement tracking”). This methodological approach allowed us to initially identify more than 70 related articles published over the past two decades. Additionally, the literature screening and review were conducted using explicit criteria tailored to the scope of our study. These criteria included the relevance of the studies to health-related domains, covering both clinical applications and healthcare. We meticulously identified and selected literature that specifically focused on NET technologies, as opposed to studies involving remote eye tracking where the distance exceeds 10 cm. Following this stringent selection process, we performed a more focused review of 25 articles from the past two decades with the details of devices and features. An overview of wearable near-eye tracking technologies for health is shown in Figure 1.

## 2. State of the Art in Wearable NET Technologies

As outlined in Section 1, non-invasive eye-tracking techniques can be classified by their signal sources into VOG, IOG, and EOG. We herein explore the underlying principles, distinctive characteristics, and medical benefits of these technologies.

### 2.1. Video Oculography

A VOG setup comprises a video camera that records eye movements using either visible or infrared light coupled with a computer that stores and analyzes the gaze data [23].

**Figure 2:**
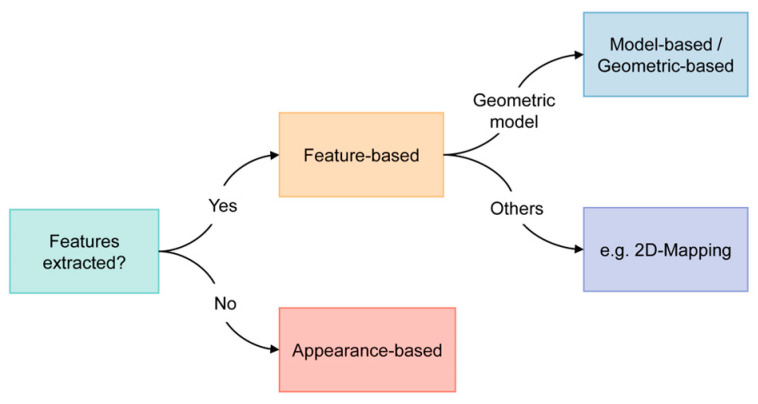
Classification criteria of wearable NET technologies discussed in this review.

Based on existing reviews and literature, there are three main categories for methods in VOG: the feature-based, appearance-based, and model-based methods. However, our review revealed that the definitions and boundaries between these concepts are somewhat ambiguous, and these methods are often used in conjunction to fully leverage the acquired image or video data. Given this overlap and to streamline the classification, we propose categorizing these methods into two distinct groups: feature-based and appearance-based (Figure 2). It is noteworthy that the majority of the studies we reviewed employed the feature-based approach.

Feature-based eye tracking relies on identifying and tracking specific features or landmarks in the eye, which are often reflected by intensity levels or intensity gradients [13]. This method is often precise and robust, but it may require careful calibration and is sensitive to lighting conditions and occlusions. In contrast, appearance-based eye tracking focuses on capturing and analyzing the overall appearance of the eye, which is more robust for visual disturbances, making it more suitable for real-world applications. However, it may require a larger amount of training data and computational resources.

#### 2.1.1. Feature-Based Eye Tracking

The initial stage in featured-based eye tracking is to extract relevant features, which often include pupil size, saccade, fixations, velocity, blink, and pupil position [33]. The extracted eye features are then used for gaze point calculation. 

A number of NET studies investigate differences in participant groups using VOG, such as in [15], which validated the variance in visual control strategies between experts and beginners in the virtual display of urethral prostatectomy. In [34], the very first study to analyze visual gaze during actual Esophagogastroduodenoscopy, gaze patterns were detected using heatmaps, and metrics such as observation time, fixation duration, and the FD-to-OT ratio were obtained. This study provides suggestions for specific visual gaze patterns of endoscopists in real practice, which might have potential applications in medical education and training. Another study revealed that VOG can differentiate the visual gaze patterns between experienced and novice endoscopists, highlighting its potential as a powerful training tool for novice colonoscopists [35]. The analysis of gaze patterns provided insights into why adenomas are often overlooked at the hepatic flexure during colonoscopy. By establishing efficient search patterns and minimizing variability in adenoma detection rates, this study lays the groundwork for improving colonoscopy training and performance.

Other features used in disease-related studies include motion velocity and acceleration, as well as average viewing times, contrast, and saliency values for fixations made to the different regions, which can be computed via custom software [36]. Additionally, a study utilizing WearCam—a wearable wireless camera—monitored focused attention in young children during play [37]. This VOG method captures gaze direction and duration to analyze attention patterns as well as color detection and face detection, potentially enabling the early detection of attention-related disorders such as autism.

- Geometric-based method

**Figure 3:**
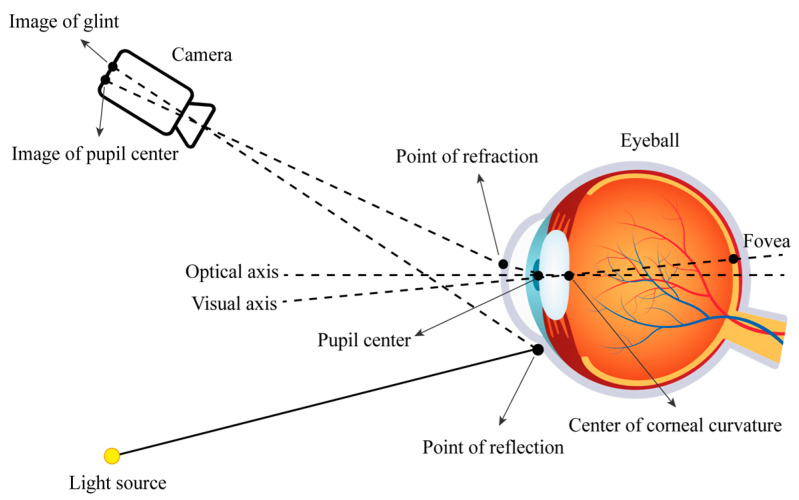
Diagram of the camera, eyeball, and light source.

The eye tracking method based on the geometric human eye model estimates the gaze direction of a 3D coordinate by relying on invariant facial features [38]. The gaze point of the human eye is estimated by the obtained line of sight direction vector and information in the scene [39]. A schematic diagram illustrating the simulation is shown in Figure 3. 

The application of geometric-based methods in medical and healthcare fields is relatively limited due to the complexity of the models and their potential lack of generalizability, making them less suitable for widespread clinical use. However, these methods are more applicable and prevalent in research focused on eye diseases and specialized surgical applications, where detailed anatomical modeling is crucial.

- Others (non-geometric based method)

**Figure 4:**
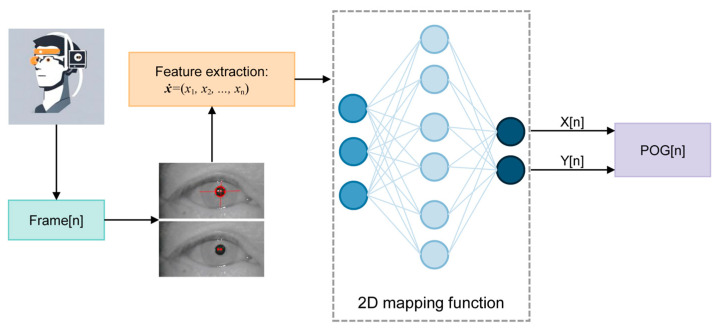
Diagram of a standard eye tracker with a 2D mapping method. (POG: point of gaze) Adapted with permission from [40].

While we have previously discussed the model-based method and its drawbacks, mapping methods provide an advantageous alternative. These methods are simpler to implement, do not require additional hardware calibration, and allow for quicker setup, which greatly enhances user convenience [40]. Consequently, most commercial gaze tracking systems opt for 2D mapping feature-based methods with IR cameras and active IR illumination to ensure precise gaze estimation, as shown in Figure 4.

**Figure 5:**
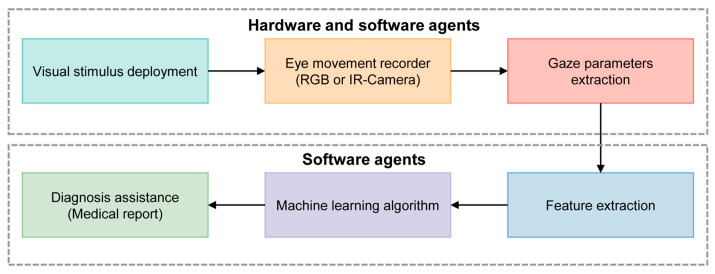
Scheme of the ML concept applied to the diagnosis of cognitive impairment using an automatic video-oculography register. Adapted with permission from [41].

Figure 5 shows a typical processing pipeline for the diagnosis of cognitive impairment using machine learning (ML) algorithms. Initially, visual stimuli are deployed to provoke eye movements, which are then captured by a camera acting as an eye movement recorder. The software then analyzes these metrics to identify patterns indicative of cognitive impairments. ML algorithms further examine these features to detect abnormalities. The findings are then compiled into a detailed diagnostic report.

#### 2.1.2. Appearance-Based Eye Tracking

The development of ML algorithms in computer vision has facilitated the emergence of appearance-based approaches in gaze estimation. Different from analytical models, these methods rely on large datasets and statistical models to construct the mapping function [23]. As a consequence, they require sufficient data rather than a deep understanding of intrinsic theories.

Appearance-based eye tracking directly analyzes raw eye images captured by cameras, treating gaze estimation as an image regression [42]. Appearance-based methods offer notable advantages in their capacity to manage intricate image features and cope with variations in lighting conditions [43]. This shift towards data-driven techniques allows for more flexible and potentially more reliable assessments in diverse patient populations and environments, as these methods do not require an in-depth theoretical understanding but rather depend on the availability of extensive training data to refine their accuracy and robustness. Nevertheless, this method is resource-intense and could encounter scalability issues, such as limitations in accommodating variations in head pose and other cerebral factors.

### 2.2. Infrared Oculography

**Figure 6:**
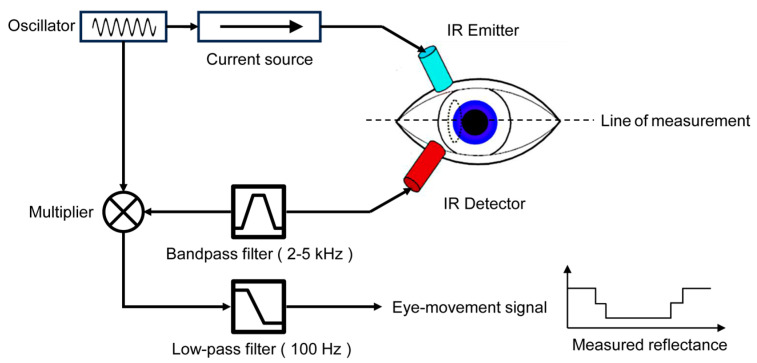
Eye movement measurement using IROG, adapted with permission from [44].

IOG is an eye-tracking method that measures the intensity of infrared light reflected from the sclera, the white part of the eye, to gather information about eye position. This method often involves the use of a wearable device, such as a pair of glasses equipped with an infrared light source. The IR light source illuminates the eye, and the changes in the reflected light can be captured with detectors and analyzed to determine eye movement and position, as shown in Figure 6.

IOG is particularly advantageous in environments such as varying light or low light conditions, leveraging infrared light which is “invisible” to the human eye and thus non-distracting to subjects. The resilience of this technology to ambient lighting variations ensures reliable measurements regardless of external light conditions. Its unobtrusiveness and accuracy also extend its utility to scenarios such as driving fatigue monitoring [45], and neuroscience [46], where natural behavior and uninterrupted observation are critical.

In one study, researchers utilized both 3D and 2D methods to analyze the gaze patterns of patients suffering from Superior Oblique Myokymia [47]. Patients were asked to maintain a primary gaze and to look in various eccentric gazes, while also measuring saccade amplitudes and velocities. The results highlight the potential of IOG in better understanding Superior Oblique Myokymia and suggest that specific medications might help manage symptoms, offering new avenues for treatment and developing effective therapies. Besides gaze patterns, facial patterns could also be recorded together, such as in [48], which leveraged IOG technology to investigate facial visual attention deficits in individuals with schizophrenia, identifying specific patterns of fixations and saccades. Another study utilized a portable real-time IOG monitoring system measuring lids, iris, and blinks to enhance the clinical diagnosis of eyelid ptosis [49]. By employing infrared eye-tracking technology, the system captured key features such as blink patterns and eyelid behaviors in real time. These metrics provided novel diagnostic markers for myasthenia gravis patients, offering new avenues for clinical investigations into various eyelid movement disorders.

### 2.3. Electrooculography

**Figure 7:**
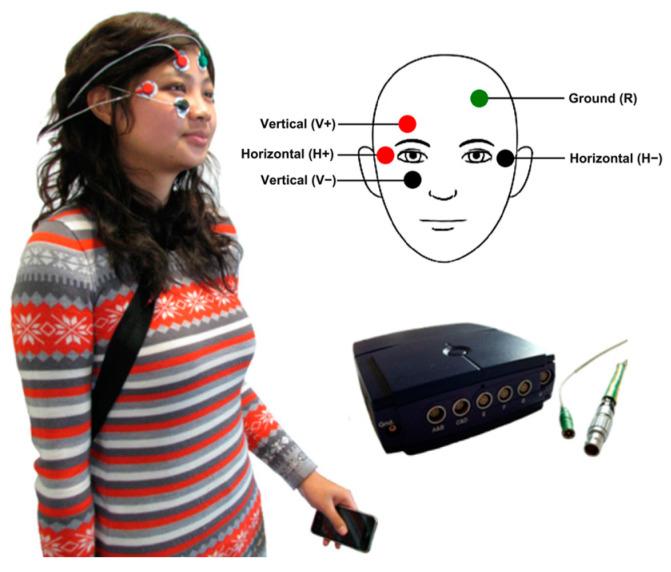
Examples of EOG: The wearable system developed in [50]. Placement of the EOG electrodes on the head and shows the iPhone and the Mobi8 device carried by the user. Electrode placement was adapted with permission from [52].

EOG is a technique widely used in NET that measures the cornea-positive standing potential relative to the back of the eye, typically the retina. An EOG system captures these changes in electric potentials using electrodes placed around the eyes—typically above and below the eye for vertical movements, and on the sides for horizontal movements, as shown in Figure 7. These voltage differences can be translated into data that indicate the direction and amplitude of eye movements. 

EOG is particularly useful for tracking eye movements over long periods, as it is less susceptible to external lighting conditions compared to other eye-tracking methods such as VOG-based systems. This makes EOG valuable in various applications, from mental monitoring [50] and neurological research [51] to user interface design and motor rehabilitation [20].

For enhanced signal quality, an innovative eye-tracking system was developed for real-time 3D visualization of eye and head movements. This system features magneto-resistive detectors mounted on the patient’s head and includes a small magnet embedded in a contact lens [51]. Its efficacy highlights its potential for advancing neurological research and improving patient care through continuous monitoring capabilities. However, the system’s reliance on specialized equipment and the need for precise calibration may limit its accessibility and necessitate regular maintenance, posing challenges for widespread medical adoption. 

EOG is often used in conjunction with other eye-tracking technologies such as IOG or VOG, including remote infrared eye-tracking systems, as demonstrated in [20]. In this particular study, the eye movements of older adults and individuals with Parkinson’s disease were accurately monitored. The setup included a wireless mobile EOG system to record horizontal saccades, a head-mounted mobile eye tracker for general saccadic recording, and a dual camera system combining a monocular infrared eye camera and a fish-eye field camera for precise pupil localization. The high temporal resolution of 1000 Hz provided by the EOG system effectively compensates for the lower tracking frequency of 50 Hz from the IR eye-tracker, ensuring detailed and responsive tracking of rapid eye movements essential for accurate analysis in clinical research. There are four stages involved in processing raw EOG voltage signal data with additional information from IOG and VOG [20]:

- **Preprocessing:** This initial stage includes baseline offset removal to adjust the starting point of the EOG signal to a standard reference, followed by filtering and noise removal to clean the data for accurate analysis;
- **Calibration and peak detection:** The signal is then calibrated to convert the raw EOG data into meaningful measurements that correspond to eye movements. This involves creating a calibration conversion factor that aligns the electrical signals with actual eye movement degrees. Following this, the system detects peaks corresponding to left or right eye movements that exceed 5 degrees;
- **Eye movement detection:** establishes velocity and acceleration thresholds to categorize different types of eye movements. This includes detecting saccades (rapid movements) with specific velocity and acceleration criteria, fixations (steady gaze) with lower velocity and longer duration thresholds and blinks characterized by very high velocity and acceleration;
- **Quantification of eye movement events:** Finally, the processed data are classified into specific events based on velocity, acceleration, and duration parameters: for saccades, the system measures the number, frequency, distance, duration, direction, and timing; for fixations, it records the number, duration, and timing.

Another study utilized a combination of EOG and VOG systems, both of which are portable and support real-time data processing, making them suitable for daily use in uncontrolled environments [50], as shown in Figure 7. This integration has led to high accuracy in capturing critical eye movement characteristics, such as saccades and smooth pursuits, which are vital for mental health monitoring.

EOG can also be integrated with electroencephalography (EEG) to simultaneously measure eye movements and brain activity, which avoids synchronization challenges that can arise with separate systems. In [53], electrodes were strategically placed in periocular regions to capture horizontal and vertical eye movements and were complemented by head stabilization techniques for EEG recording. This combined methodology is especially valuable for examining fixation- and saccade-related neural potentials, offering insights into the underlying neural mechanisms that govern eye movement control. This approach not only simplifies the experimental setup but also enriches the quality of data for advanced neuroscientific research.

## 3. Applications of NET for Health

Table 2 summarizes various applications of typical NET sensors in several health-related fields. Many studies rely on well-established commercial NET sensors. Compared with IOG and EOG, VOG is being used more widely in health-related domains. As a newly developed technology, NET is now primarily used in the areas of endoscopy and mental health monitoring.

### 3.1. NET in Endoscopy

**Figure 8:**
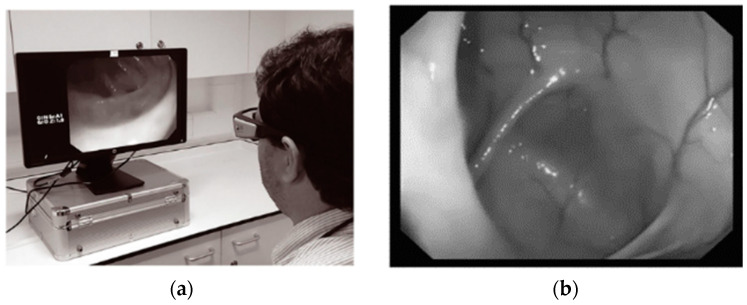
(**a**) User wearing eye tracking glasses observing withdrawal video. (**b**) Hepatic flexure on the left side of the screen. Endoscopic application [35].

The examination of visual patterns exhibited by endoscopists during colonoscopy procedures is an interesting area of endoscopy with an evolving evidence base [65]. The visual gaze patterns of endoscopists are paramount in the detection of colonic pathology. The eye movements of an endoscopist during a colonoscopy can be assessed by gaze analysis. Research using gaze analysis is allowing us a greater insight into how visual patterns differ between experts with higher ADR and nonexperts with lower detection rates [30], as shown in Figure 8.

NET has the potential to provide a more objective measure for detecting lesions during endoscopy. This method is more valuable than relying on subjective feedback from endoscopists, particularly when accessing new technologies for improving adenoma or bleeding vessel detection [34]. Eye-tracking glasses can also be used as a new steering system for endoscopes, allowing endoscopists to have bimanual freedom for instrumentation [54]. The application of gaze analysis and control in endoscopy presents exciting potential for advancing the field and its crucial role in mitigating the growing global burden of gastrointestinal cancer [2]. Yet, given that gaze analysis represents a recent and novel field of research, the existing studies are limited to small sample sizes and yield inconclusive results.

### 3.2. NET in Mental Health Monitoring

Research in experimental psychology and clinical neuroscience has demonstrated a significant correlation between eye movements and mental disorders [66,67]. In the past, diagnostics based on eye movement were limited to controlled laboratory settings; however, wearable eye trackers now enable continuous monitoring and analysis of eye movements [68]. As a delicate function connected to the central nervous system, eye motricity is susceptible to disturbances arising from disorders and diseases affecting various brain regions such as the cerebral cortex, brainstem, or cerebellum. Analysis of resultant eye movement dysfunctions provides valuable insights into the localization of brain damage [69] and serves as a reliable marker for dementia and numerous other brain-related conditions [66,70]. 

Take schizophrenia as an example: it is a mental disorder characterized by antisocial personality disorder. Previous studies have proven that schizophrenia impairs smooth pursuit [71] and increases the frequency of saccades, especially catch-up saccades during smooth pursuit [72]. Despite over 35 years of investigation into eye movement impairments in schizophrenic patients, this remains an active area of research, with ongoing efforts aimed at developing portable and cost-effective devices for further studies [73]. Holzman and Levy [74] used EOG for its portability, despite acknowledging that it may be less precise than video-based trackers at the time of writing. Their findings reveal that smooth pursuit impairment not only in schizophrenia but also in psychotic patients. They demonstrated two distinct types of smooth pursuit impairment: (1) pursuits replaced by rapid eye movements or saccades; and (2) small amplitude rapid movements intruding pursuit, leaving the shape intact but having a cogwheel appearance. Furthermore, they proposed that smooth pursuit impairment may qualify as a genetic indicator of the predisposition for schizophrenia. In addition, non-smooth pursuit records are also found in the close family of schizophrenic patients, and a good number of psychotic patients without schizophrenia are found to have bad smooth pursuit eye movements, too [74]. 

### 3.3. NET + X for Health

NET can be utilized not only as a standalone tool but also in conjunction with other technologies to enhance precision and accuracy or to deliver valuable supplementary insights. By integrating NET with various technologies, such as VR, EEG, or OTA, the application’s scope has been significantly expanded. This synergistic approach, referred to as NET + X, leverages multiple data sources and technological methods to improve the overall effectiveness of the system.

#### 3.3.1. NET + VR

**Figure 9:**
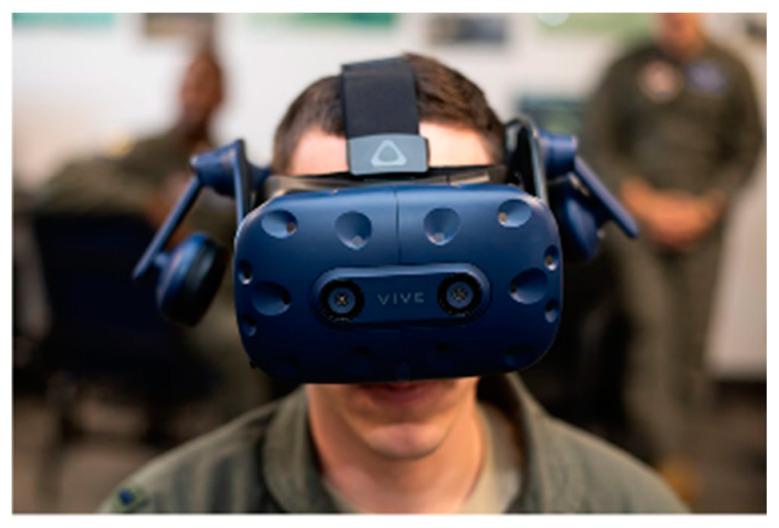
An HTC Vive Pro Eye HR HMD combined with a VOG NET sensor. Taken with permission from [25].

The fundamental principle of VR is tailoring stimuli to user actions, including head, eye, and hand movements [75]. Head-mounted display (HMD)-based VR relies on the accurate tracking of head movements to synchronize visual scene motion with head movement, facilitated by advancements in head-tracking technology. Anticipated advancements in HMD-based eye-tracking technology, as shown in Figure 9, will allow for fundamental advances in VR applications based on eye movement [25].

Eye-tracking technology VR has various applications in the clinical context, including for diagnostic, therapeutic, and interactive purposes [76]. Traditionally, neuro-ophthalmic diagnosis has been conducted in a very basic manner at the patient’s bedside [77]. Fortunately, this process could be greatly improved by the development of uniform HMD-based diagnostic tools that have precise stimulus control to elicit specific and relevant eye movements, such as pursuit, saccades, nystagmus, etc. For example, when doctors wear VR headsets, the patient’s body model is reconstructed in a virtual VR operating room, allowing the doctor to observe the organs or lesions in a 360° view and make more accurate preliminary measurements and estimates of the affected areas. This enables the doctor to develop more reasonable, accurate, and safer surgical implementation plans [78].

Nonetheless, most current clinical VR equipment for eye tracking uses commercial devices, often unsuitable for clinical use [79]. For example, Zhu et al. [80] mention that most HMDs have to be modified by removing, enclosing, or replacing their textile foam and Velcro components in order to comply with clinical hygiene regulations. Most HMDs and their eye-tracking components also cannot withstand clinical disinfection procedures. Therefore, further development is necessary to achieve clinical-grade HMDs.

#### 3.3.2. NET + Other

**Figure 10:**
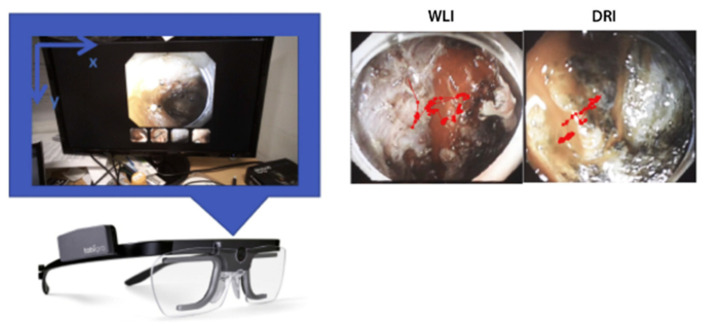
A subject wearing an eye-tracking device while searching for the bleeding point (**left**). The example of endoscopic images of the measured eye position attached to the infrared marker (**right**). WLI, White-light imaging; DRI, dual red imaging. Taken with permission from [56].

Apart from VR, there are other modalities that can be combined with NET to achieve health-related applications. In [55], an eye tracker was integrated with continuous performance tests to access patients with attention deficit hyperactivity disorder. By comparing acquired data with the health control group, this study demonstrates that eye movement measurement presented its potential to increase our theoretical understanding of attention deficit hyperactivity disorder and is beneficial for clinical decision-making. Moreover, in [56], presented in Figure 10, NET was adopted as a measurement tool to evaluate the efficacy between dual red imaging and white-light imaging for hemostasis during endoscopic submucosal dissection. The eye movements of experienced endoscopists were monitored by wearable NET glasses. The endoscopists were asked to identify bleeding points in each random video of intraoperative bleeding during endoscopic submucosal dissection. The NET glasses gave an accurate record of endoscopists’ eye movement, which also became the standard for rating the efficacy of dual red imaging and white-light imaging.

EOG-based NET could also be combined with EEG, as in [53], to study fixation- and saccade-related neural potentials and advance our understanding of the neural mechanisms involved in eye movement control, offering a robust tool for both clinical and research applications. Integrating NET with remote ET could also provide a more complete picture of eye movement behavior during various tasks, allowing for the detection and analysis of saccades in both static and dynamic conditions [20].

Another important area when using VOG NET with other techniques is motion artifact removal. One study introduced a video-based real-time eye-tracking system suitable for functional magnetic resonance imaging (fMRI) applications [54]. Interference from physiological head movement is effectively reduced by simultaneous tracking of both eye and head movements. Ref. [7] suggests that using ET technology can significantly enhance the quality of optical coherence tomography angiography (OCT-A) images by reducing motion artifacts, which is particularly problematic in patients with age-related macular degeneration.

## 4. Discussion and Conclusions

### 4.1. Summary of NET in Health

The most popular NET technologies currently include VOG, IOG, and EOG. VOG benefits from high resolution and advanced camera technology, suitable for detailed eye feature analysis and robust in real-world applications. IOG employs infrared light, effective in varying light conditions and ideal for fatigue monitoring and certain medical diagnoses. EOG measures electrical potentials around the eyes, appropriate for long-term tracking. The majority of studies reviewed employ VOG, which has benefited from recent advancements in camera technology that significantly enhance its temporal resolution. The emergence of easy-to-set-up commercial portable VOG devices emphasizes their potential for wild medical use. Conversely, though IOG and EOG are useful in certain situations, they generally yield lower resolution and are more susceptible to noise, making them less suitable for medical and research applications requiring precise eye-tracking capabilities.

Applied in endoscopy, NET can enhance medical training by differentiating visual patterns between novices and experts. As for mental health monitoring, NET is beneficial for the diagnostics of schizophrenia and dementia. Additionally, integrating NET with technologies such as VR and EEG can help clinical decision-making or improve the precision of clinical and healthcare devices.

### 4.2. Future Trend of NET in Health

#### 4.2.1. Sensor Design

Many current NET sensors have achieved wearable designs, such as in the form of glasses or head-mounted devices. Future development should focus on reducing size and weight to ensure long-term, continuous, and comfortable use for clinical and healthcare applications. Besides, the real-time transmission and computation of data are also worthy of further discussion. Since improving wearability may lead to a decrease in the speed and accuracy of real-time data transmission and computation, a balance between the pursuit of wearability, efficient real-time data transmission, and computation should be emphasized in future work.

Apart from designing more wearable NET sensors, another way to improve their versatility of applications is developing non-cooperative NET. Nowadays, NET usually relies on user cooperation, which often involves the utilization of dedicated sensors or devices. However, challenges arise in situations where cooperation is difficult, such as with infants, seniors, or individuals with disabilities. In such instances, developing methods that facilitate non-cooperative NET is imperative. Because it not only meets the requirements of different user groups but also allows NET to be applied in diverse fields and scenarios.

#### 4.2.2. Standardization

As introduced in Section 3, NET has been widely applied in endoscopy and surgery to evaluate the visual patterns of doctors to facilitate the analysis of diseases or surgical training processes. Nonetheless, currently, there is no fixed quantitative standard for evaluating the NET data obtained in various clinical cases. These data are now simply categorized using traditional evaluation scales. When summarizing and comparing various NET sensors, we found that a number of studies did not specify key parameters such as resolution, accuracy, and weight. This omission makes it difficult to quantitatively assess the measurement performance and wearability of NET sensors. With the gradual development and spread of NET technology, it is hoped that new standardized methods for NET sensors and acquired data can be developed.

#### 4.2.3. NET + X

The combination of NET and other technologies has been preliminarily applied in health-related fields. In future developments, NET can be integrated not only with clinical technologies, such as fMRI and OCT-A, but also with emerging electronic products or technologies, such as VR and AR. Since commercial VR devices are suitable for non-professionals to use, NET + VR will be developed for purposes of personal health monitoring and enhancing individual health management in daily life. Therefore, NET + VR is capable of influencing how health data are collected and utilized, eventually enabling health monitoring to be more personalized and precise.

### 4.3. Conclusions

This review provides the technical features, development, and application of health-related NET technologies. NET has already been effectively applied in several health-related fields. Meanwhile, as a relatively new technology, future efforts should focus on miniaturization and weight reduction to improve the wearable design of NET sensors. Additionally, developing non-cooperative NET methods will expand usability for groups such as infants, seniors, and individuals with disabilities. Standardizing data evaluation is essential to ensure reliable comparisons and assessments of NET systems. With further development and integration with other technologies, such as VR, AR, and fMRI, NET holds great potential to become a wearable, low-cost, high-precision tool that can be practically applied in clinical and healthcare applications.

## Figures and Tables

**Table 2:** Applications of NET sensors in health-related fields.

Ref.	Year	Application	Method	Wearability	Transmission	Device	Sampling Frequency/Hz
[9]	2014	Control of electronic wheelchair	VOG	Glasses	Wired	-	-
[14]	2016	Assessment of oculomotor abnormalities	VOG	Head-mounted	Wireless	EyeTribe	30
[15]	2014	Surgical training	VOG	Glasses	Wired	ASL Eye tracker	8.33
[20]	2017	Investigation of Parkinson’s disease	EOG + IOG +VOG	EOG: Head-mounted IOG: Head-mounted	EOG: WIFI IOG: Wired VOG: Wired	EOG: Zerowire IOG: Dikablis VOG: dual camera system	EOG: 1000 IOG: 50 VOG: 50
[35]	2016	Evaluation in colonoscopy	VOG	Glasses	Wired	Model 1.4	30
[36]	2006	Investigation of autism	VOG (infrared)	Head-mounted	Wired	EyeLink II	500
[37]	2007	Diagnosis of Developmental Disorders	VOG	Headcoil	Radio receiver	WearCam	30
[41]	2023	Detection of Hepatic Encephalopathy	VOG (infrared)	Head stabilization	Wired	Infrared Camera	100
[46]	2011	Evaluation in surgery	IOG	-	-	-	-
[47]	2017	Investigation of superior oblique myokymia	IOG	3D: Glasses 2D: Head stabilization	Wired	3D: 3-D VOG 2D: iView-X Hi-Speed	3D: 60 2D: 500
[48]	2015	Investigation of schizophrenia	VOG	Head-mounted	Wired	EyeLink II	250
[49]	2014	Diagnosis of Ocular Myasthenia Gravis	IOG	Glasses	Wired	Pupil Labs	30
[51]	2023	Oculomotor Assessment in Neurological Research	EOG	-	-	Magnetoresistive-based eye tracker	100
[53]	2019	EEG Recording	EOG	-	Wired	128-channel EGI (Electrical Geodesics, Inc., Eugene, OR, USA)	500
[54]	2007	Endoscopy	VOG	Headcoil	Wired	-	50
[55]	2022	Assessment in attention deficit hyperactivity disorder	EOG	Desktop Mount	Wired	Eye Link 1000	250
[56]	2020	Hemostasis in Endoscopy	VOG	Glasses	-	Tobii Pro Glasses 2	50
[57]	2021	Surgical training	VOG	Glasses	Wireless	Pupil Invisible	30
[58]	2007	Investigation of schizophrenia	EOG	Glasses	Wired	limbus tracker (Cambridge Research Systems, Cambridge, UK)	500
[59]	2004	Monitoring of affective state	IOG	Head Stabilization	Wired	MR-Eyetracker (Cambridge Research Systems, UK)	1000
[60]	2014	Investigation of Alzheimer’s disease	VOG	Glasses	Wired	Senso-Motoric	350
[61]	2009	Investigation of autism spectrum disorder	VOG	Head-mounted	Wired	Dual Purkinje Image eye-tracker	200
[62]	2008	Investigation of autism spectrum disorder	VOG	Head-mounted	Wired	ISCAN ETL-500	240
[63]	2013	Measurement of startle	VOG (infrared)	Head Stabilization	Wired	iView X Hi-Speed 500	500
[64]	2018	Vestibular Function Testing	VOG (infrared)	Head-mounted	Wired	Infrared Camera	30
